# Supplementary material for: Splice-Junction-Based Mapping of Alternative Isoforms in the Human Proteome
Source: Cell Rep. Author manuscript; Available in PMC 2020 Jan 15. (PMC6961840; doi:10.1016/j.celrep.2019.11.026)
Supplement: 2 [file NIHMS1546469-supplement-2.zip › DF1/PXD006675/SmoothMuscleCells/SmoothMuscleCells_5_RPS2_THTRVSVQRTQAPAVATDAR.pdf]

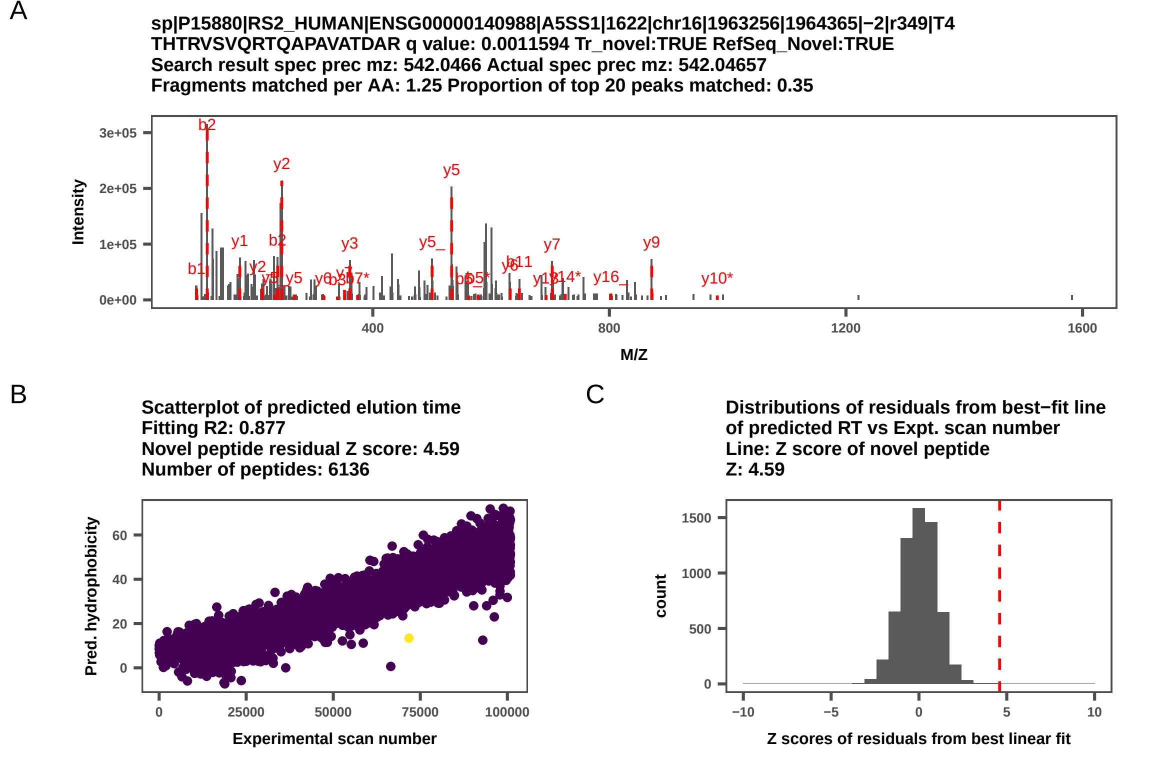
A
sp|P15880|RS2_HUMAN|ENSG00000140988|A5SS1|1622|chr16|1963256|1964365|−2|r349|T4
THTRVSVQRTQAPAVATDAR q value: 0.0011594 Tr_novel:TRUE RefSeq_Novel:TRUE
Search result spec prec mz: 542.0466 Actual spec prec mz: 542.04657
Fragments matched per AA: 1.25 Proportion of top 20 peaks matched: 0.35
Intensity
3e+05
2e+05
1e+05
0e+00
b2
y2
y5
y1
b2
y3
y5_
y7
y9
b1
y2
y5
y16_
y10*
400
800
1200
1600
M/Z
B
Scatterplot of predicted elution time
Fitting R2: 0.877
Novel peptide residual Z score: 4.59
Number of peptides: 6136
Pred. hydrophobicity
60
40
20
0
0
25000
50000
75000
100000
Experimental scan number
C
Distributions of residuals from best−fit line
of predicted RT vs Expt. scan number
Line: Z score of novel peptide
Z: 4.59
count
1500
1000
500
0
−10
−5
0
5
10
Z scores of residuals from best linear fit
